# Impact of co-infections with enteric pathogens on children suffering from acute diarrhea in southwest China

**DOI:** 10.1186/s40249-016-0157-2

**Published:** 2016-06-27

**Authors:** Shun-Xian Zhang, Yong-Ming Zhou, Wen Xu, Li-Guang Tian, Jia-Xu Chen, Shao-Hong Chen, Zhi-Sheng Dang, Wen-Peng Gu, Jian-Wen Yin, Emmanuel Serrano, Xiao-Nong Zhou

**Affiliations:** National Institute of Parasitic Diseases, Chinese Center for Disease Control and Prevention, Shanghai, 200025 People’s Republic of China; Key Laboratory for Parasite and Vector Biology, Ministry of Health of China, WHO Collaborating Center for Tropical Diseases, National Center for International Research on Tropical Diseases, Shanghai, 200025 People’s Republic of China; Yunnan Provincial Center for Disease Control and Prevention, Kunming, People’s Republic of China; Centre for Environmental and Marine Studies, Departamento de Biología, Universidade de Aveiro, Aveiro, Portugal; Servei d’Ecopatologia de Fauna Salvatge, Departament de Medicina i Cirurgia Animals, Universitat Autònoma de Barcelona, Bellaterra, Spain

**Keywords:** Co-infection, Diarrhea, Bacteria, Virus, Intestinal protozoa, Children, Severity, Kunming, Yunnan, China

## Abstract

**Background:**

Acute diarrhea is a global health problem, resulting in high morbidity and mortality in children. It has been suggested that enteric pathogen co-infections play an important role in gastroenteritis, but most research efforts have only focused on a small range of species belonging to a few pathogen groups. This study aimed to assess the impact of co-infections with a broad range of enteric pathogens on children aged below five years who suffer from acute diarrhea in southwest China.

**Method:**

A total of 1020 subjects (850 diarrhea cases and 170 healthy controls) were selected from four sentinel hospitals in Kunming, Yunnan province, southwest China, from June 2014 to July 2015. Stool specimens were collected to detect five virus (rotavirus group A, RVA; norovirus, NoV; *Sapovirus,* SaV; astrovirus, As; and adenovirus, Ad), seven bacterial (diarrheagenic *Escherichia coli*, DEC; non-typhoidal *Salmonella*, NTS; *Shigella* spp.; *Vibrio cholera*; *Vibrio parahaemolyticus*; *Aeromonas* spp.; and *Plesiomonas* spp.), and three protozoan (*Cryptosporidium* spp., *Giardia lamblia*, and *Blastocystis hominis*, *B. hominis*) species using standard microbiologic and molecular methods. Data were analyzed using the partial least square regression technique and chi-square test.

**Results:**

At least one enteric pathogen was detected in 46.7 % (*n* = 397) of acute gastroenteritis cases and 13.5 % (*n* = 23) of healthy controls (χ^2^ = 64.4, *P* < 0.05). Single infection with RVA was associated with acute diarrhea (26.5 % vs. 5.8 %, *P* < 0.05). The prevalence of a single infection with *B. hominis* in diarrhea cases was higher than in healthy controls (3.1 % vs. 0.5 %, *OR* = 4.7, 95 % *CI*: 1.01–112.0). Single infection with NoV GII was not associated with diarrhea (4.4 % vs. 3.5 %, *OR* = 1.2, 95 % *CI*: 0.5–3.3). Single infections with bacterial species were not observed. The prevalence of co-infections with two enteric pathogens in diarrhea cases was higher than in asymptomatic children (20.1 % vs. 5.3 %, *P* < 0.05). RVA-NoV GII was the most common co-infection in symptomatic children (4.4 %), with it aggravating the severity of diarrhea.

**Conclusions:**

Although it is clear that RVA has an overwhelming impact on diarrhea illnesses in children, co-infection with other enteric pathogens appears to also aggravate diarrhea severity. These findings should serve as evidence for public health services when planning and developing intervention programs.

**Electronic supplementary material:**

The online version of this article (doi:10.1186/s40249-016-0157-2) contains supplementary material, which is available to authorized users.

## Multilingual abstracts

Please see Additional file 1 for translations of the abstract into the six official working languages of the United Nations.

## Background

Despite that mortality caused by diarrhea has sharply decreased in the past three decades [1], gastroenteritis is still the most common health problem worldwide. About 63 % of all diarrhea cases globally occur in children below five years of age [1, 2], and diarrhea is the second cause of infant death in low-income countries. More than 70,000 deaths are attributed to acute diarrhea every year [2]. According to the World Health Organization (WHO), diarrhea causes more deaths in children than malaria and HIV/AIDS combined [3].

Several virus, bacterial, and protozoan species are etiological agents of acute diarrhea [4, 5]. Among them, rotavirus group A (RVA) is the most common cause of gastroenteritis in countries that do not have RVA vaccination programs. As a result, 192,770 children and 25,000 adults die every year due to RVA infections [2, 6]. But other viruses such as norovirus strain GII (NoV GII) are also involved in gastroenteritis outbreaks worldwide [7, 8]. NoV is considered a common foodborne viral disease causing around 18 % of all diarrhea cases in outpatient and inpatient settings among all age groups [9, 10].

Diarrheagenic *Escherichia coli* (DEC) is frequently associated with diarrhea [11–13]. DEC is classified into several subtypes based on pathogenic mechanisms and clinical features. These include enteropathogenic *E. coli* (EPEC), enterotoxigenic *E. coli* (ETEC), enteroaggregative *E. coli* (EAEC), enteroinvasive *E. coli* (EIEC), enterohemorrhagic *E. coli* (EHEC), and diffusely adherent *E. coli* (DAEC). Although considered to be a common foodborne pathogen in regions with poor sanitation and water scarcity [14], DEC are not routinely screened for in most countries, because of the difficulty of implementing the technically complex laboratory protocol [15]. Bacterial species such as non-typhoidal *Salmonella* spp. (NTS) are other leading causes of bacterial diarrhea worldwide, resulting in 93,800,000 cases of gastroenteritis and 155,000 deaths globally each year [16–18].

But not only viruses and bacteria cause diarrhea in tropical and subtropical regions with poor sanitation [19, 20]—protozoan species are other culprits. Although protozoan diarrhea has traditionally been considered to account for a small number of acute diarrhea cases, recent research has shown that enteric protozoan pathogens, including *Cryptosporidium* spp., are the second biggest causes of gastroenteritis and deaths in infants and toddlers [21]. In fact, around 100,000 deaths due to gastroenteritis worldwide are attributable to protozoan species [6].

The previously mentioned enteric pathogens, however, are also found in asymptomatic people, which make it difficult to link gastroenteritis to a specific etiological agent. For example, RVA prevalence is around 18 % in diarrhea cases but 3 % in healthy people in Thailand [22], and the detection rate of ETEC can be 34.0 % in symptomatic patients but 29.0 % in asymptomatic controls in Côte d’Ivoire [23]. Meanwhile, co-infection by multiple groups of enteric pathogens is demonstrated to be the norm in diarrhea cases and asymptomatic controls. At least two enteric pathogens were detected in 37 symptomatic patients (54.0 %) and in 47 healthy controls (69.0 %) in Dabou, southern Côte d’Ivoire [23]. It has been suggested that it is important to consider enteric pathogen communities rather than just single pathogens when assessing the impact of co-infections in diarrhea cases [24, 25].

In general terms, people who are co-infected by multiple groups of pathogens are often considered to be in poorer health than people who have single infections [26]. Co-infection can also increase treatment costs, probably as a result of clinical complications due to interactions among co-infecting pathogens [27]. Thus, according to the most recent trends in infectious diseases research [28], studies oriented at investigating the role of co-infections with enteric pathogens in people with acute diarrhea are now required more than ever.

Seasonal variations in the incidence of acute diarrhea are known globally. In some parts of the world, fecal-oral disease transmission is linked to the use of ground and well water that increases outside of the wet season [29]. In fact, in mild subtropical climates, incidence of diarrhea appears to decrease after the rains begin. Seasonal variations in occurrence have been described for all types of enteric pathogens linked to gastroenteritis: protozoan, bacteria, and viruses [30–33].

In China, diarrhea is still one of the most serious public health problems, especially in low-income regions such as southwest and northwest China. More than 10,000 children die from diarrhea in China annually [34], while it has been reported that more than 70,000,000 patients suffer from diarrhea annually, with an incidence of up to 55.9/10,000,000. This high number of diarrhea cases has resulted in diarrhea being listed by China Information System for Diseases Control and Prevention as one of the top three among 39 notifiable infectious diseases [11, 35]. However, the burden of diarrhea caused by enteric pathogens is not accurately estimated due to a lack of diagnostic capacity in most medical institutions in China. In fact, only RVA is routinely detected in most hospital laboratories [36]. As a result, pathognomonic agents in patients were detected only in 5 % of the diarrhea patients reported in 2010 year [11]. In addition, most of the diarrhea studies have been limited to either bacterial or viral etiologies [11, 37, 38].

Hence, an understanding of the role of co-infection with enteric pathogens including virus, bacterial, and intestinal protozoan species is now a priority in global health programs for the eradication of acute diarrhea in children. In this study, taking advantage of the intensive survey on the etiology of acute diarrhea carried out by four sentinel hospitals in Kunming, southwest China, we investigated the impact of co-infection with seven virus, seven bacterial, and three protozoan species on children aged below five years suffering from diarrhea. Our study had two aims: 1) to explore the impact of concomitant infections on the severity of acute diarrhea, and 2) to assess whether enteric pathogens in sick children co-occur by chance or follow a pattern.

## Methods

### Definitions of diarrhea cases and healthy controls

In accordance with the WHO’s definition of diarrhea [39], a case of acute gastroenteritis was defined as a person who had more than three episodes of abnormal stool within 24 h (e.g. loose, watery, bloody, or mucousy stool) for any period lasting for <2 weeks. A healthy control was defined as a person who did not have any diarrhea symptoms in the seven days prior to recruitment into the study.

### Study site

The study was conducted from July 2014 to June 2015 in Kunming (25° 02’ 20”N, 102° 43’05” E, 1892 m above sea level), Yunnan province, southwest China. Kunming has a humid subtropical climate characterized by mild (mean temperature = 11.4 °C, min = 8 °C, max = 15 °C) and dry (mean precipitation = 33.4 mm, min = 12 mm, max = 89 mm) falls and winters. Spring and summers are also mild (mean temperature = 23 °C, min = 19 °C, max = 29 °C), but are wet (mean precipitation = 159.6 mm, min = 92 mm, max = 206 mm). During the study period, four sentinel hospitals were selected: the Pushan Health Center in Kunming, the First Affiliated Hospital of Kunming Medical University, the First People’s Hospital of Yunnan Province, and Kunming Children’s Hospital.

### Collection of stool specimens and basic information

1020 subjects (850 diarrhea cases and 170 healthy controls) were recruited into this study. Each stool specimen was collected with sterile sampling cups using sterile cotton swabs, and then was transferred to the laboratory of Yunnan Provincial Center for Disease Control and Prevention in Cary Blair Transport Medium (Oxoid Ltd, Basingstoke, UK) within 12 h. A structured questionnaire was used to elucidate the following information from each diarrhea case and healthy control after the stool samples were collected: clinical manifestations associated with diarrhea (e.g. vomiting, fever, and/or dehydration), demographic data (age, sex, and residence), and types of stool samples (watery, mucousy, or bloody, or other form).

### Assessing the severity of diarrhea

The definition of diarrhea severity is less precise and depends on clinical signs such as dehydration or dysentery. In this study, we considered both the maximum number of liquid stools (MNLS) and the maximum number of bloody stools (MNBS) in a 24 h period as proxy for gastroenteritis severity. Though recent studies have suggested the usefulness of scores based on selected clinical manifestations to assess diarrhea morbidity [40], the maximum number of stools per 24 h period is still one of the main ways used to assess the severity of diarrhea. In fact, recent research has shown that the MNLS is highly correlated with the degree of anorexia, fever, vomiting, and dehydration due to diarrhea [30].

### Detection of enteric pathogens

Stool samples were divided into three aliquots for the detection of virus (RVA; NoV GI; NoV GII; sapovirus, SaV; astrovirus, As; and adenovirus, Ad), bacterial (DEC, *Shigella* spp., NTS, *Vibrio cholera*, *V. parahaemolyticus*, *Aeromonas* spp., and *Plesiomonas* spp.), and protozoan (*Cryptosporidium* spp., *Giardia lamblia*, and *Blastocystis hominis*, *B. hominis*) species.

#### Detection of virus species

Each stool sample was prepared as a 10 % weight/volume suspension to extract viral nucleic acid. Deoxyribonucleic acid (DNA) or ribonucleic acid (RNA) was extracted from each sample using the appropriate kits (Geneaid Biotech Ltd, Taiwan, China), following the manufacturer’s instructions. For four RNA viruses, namely RVA, NoV GI, NoV GII, SaV, and As, complementary DNA (cDNA) was synthesized using a random primer (Takara Bio Inc, Shlga, Japan) at 55 °C for 1.5 h, followed by 100 °C for 10 min, and holding at 4 °C. The reverse transcriptase polymerase chain reaction (RT-PCT) method was used to detect the presence of RVA in the stool, as described previously [41]. Multiplex RT-PCR was used to detect the presence of NoV GI, NoV GII, and SaV, as also previously described [42, 43]. Meanwhile, RT-PCR was used to detect As, based on a previous study [44], and presence of Ad was directly detected using PCR [38]. All suspicious positive viral pathogens found using agarose gel electrophoresis were determined to be positive with sequencing.

#### Detection of bacterial species

Several methods to detect bacterial pathogens were used including the serological method, germiculture, biochemical identification, and molecular biology technology such as PCR (see Table 1). Each stool sample was plated directly onto MacConkey agar (MAC, Oxoid Ltd, Basingstoke, UK) to detect the presence of DEC, which included the subtypes EAEC, EPEC, EAEC, EHEC, and ETEC, at 37 °C for 18 h. Then, 10 putative DEC colonies were selected from the MAC plates and mixed with 150 μl of water without deoxyribonuclease (DNase) and ribonuclease (RNase) in order to extract the genome using the hot extraction method (100 °C for 10 min). Then, the real-time quantitative PCR (qPCR) method was used to detect the five subtypes with specific primers, as described previously [45, 46]. The final reaction volume was 20 μl, consisting of 1 μl forward primer (10 μmol), 1 μL of reverse primer (10 μmol), 1 μl of DNA template, 7 μl of water and 10 μl PCR master mix (Takara Bio Inc, Shlga, Japan). The cycling condition for each subtype of DEC was: 95 °C for three minutes, 40 cycles at 95 °C for five seconds, and 60 °C for 30 s, with the fluorescence recorded at the annealing stage. Specific details on detecting the presence of DEC have been previously described elsewhere [11, 12, 45, 46]. Each stool was inoculated into selenite brilliant green sulfa enrichment broth (Oxoid Ltd, Basingstoke, UK) at 37 °C for 18 h, and was then plated onto Salmonella Shigella agar (Oxoid Ltd, Basingstoke, UK) and xylose lysine deoxycholate agar (Oxoid Ltd, Basingstoke, UK) to detect NTS and *Shigella* spp. strains, after an 18 h incubation at 37 °C. The suspicious colony was plated onto CHROMagar™ Salmonella medium (CHROMagar, Paris, France) and cultivated at 37 °C for 18 h. For suspected colonies of NTS and *Shigella* spp., the systematic biochemical identification of suspected positive strains was performed using the VITEK® 2 Compact instrument (bioMerieux, Marcyl’Etoile, France). When NTS and *Shigella*.spp were determined to positive, then, further serological testing was used to isolate their subtype.

**Table 1:** Diagnostic techniques used to detect enteric pathogens in 1020 children aged below five years in Kunming, southwest China

Enteric pathogens	Diagnostic technique					Reference	Comments
	Culture	Biochemical	Serological	PCR	Microscopy		
Virus							
RVA				√		[41]	NoV was divided into GI and GII strains. RVA, NoV, SaV, and As were RNA viruses detected using RT-PCR. Ad was a DNA virus detected using PCR.
NoV				√		[42, 43]	
SaV				√			
As				√		[44]	
Ad				√		[38]	
Bacteria							
DEC	√			√		[11, 12, 45, 46]	DEC involved EAEC, EPEC, EIEC, EHEC, and ETEC types. All were detected using qPCR.
NTS	√	√	√			[11, 12]	
*Shigella* spp.	√	√	√				
*Vibrio cholera*	√	√	√				
*Vibrio parahaemolyticus*	√	√	√				
*Aeromonas* spp.	√	√	√				
*Plesiomonas* spp.	√	√	√				
*Protozoa*							
*B. hominis*				√		[47]	*B. hominis* was detected using PCR, whereas *Cryptosporidium* spp. and *Giardia lamblia* were detected using nested PCR.
*Cryptosporidium* spp.				√		[48, 49]	
*Giardia lamblia*				√			

(Note: The “√” symbol indicates the diagnostic techniques applied in this study)

Each stool sample was directly inoculated onto alkaline peptone water (Oxoid Ltd, Basingstoke, UK) at 37 °C for 18 h to examine for *Vibrio cholera*, *Vibrio parahaemolyticus*, *Aeromonas* spp., and *Plesiomonas* spp., and was then plated onto thiosulfate-citrate-bile salts-sucrose agar (Oxoid Ltd, Basingstoke, UK) at 37 °C for 18 h. Suspicious colonies were selected to conduct the oxidase experiment. If the oxidase test resulted in a positive reading, the systematic biochemical identification for these suspicious colonies was confirmed using the VITEK® 2 Compact instrument (bioMerieux, Marcyl’Etoile, France), the procedure for which has been described previously [11, 12].

#### Detection of intestinal protozoan species

Nucleic acid of intestinal protozoan pathogen was extracted from each stool specimen with TIANamp Stool DNA Kit (Tiagen Biotech Ltd, Beijing, China), according to the manufacturer’s instructions. It was amplified by conventional PCR for *B. hominis* [47], and nested PCR for *Cryptosporidium* spp. and *Giardia lamblia* [48, 49].

### Statistical modeling

#### Case-control comparisons

Prevalences at 95 % confidence intervals (CIs) for single infections and co-infections in both diarrhea cases and controls were calculated using epiR library version 0.9–74. Odds ratios (*OR*s) at 95 % were calculated using EpiTools library version 0.5–7 [50]. Prevalences were compared using the chi-square or Fisher’s exact tests. All statistical analyses were performed using R version 3.3.0 [51].

#### Assessing the impact of co-infection with enteric pathogens on diarrhea severity

The partial least square (PLS) regression method was used to assess the impact of co-infection with enteric pathogens on the severity of acute diarrhea among children. The PLS technique is probably the least restrictive of the multivariate techniques for exploring complex ecological patterns [52], including the impact of co-infections on the host’s health [53]. In addition, PLS is distribution free and well suited to deal with multicollinearity [54].

In our analysis, we defined explanatory and response components or blocks. The explanatory block (PLS X’s component) was defined by a presence-absence matrix [55] representing the enteric pathogen community of the virus (Ad, As, RVA, NoV GI, and NoV GII, and SaV), bacterial (DEC, NTS, *Shigella* spp., *Aeromonas* spp., *Plesiomonas* spp., *Vibrio cholerae* and *Vibrio parahaemolyticus*), and protozoan (*B. hominis*, *Cryptosporidium* spp., and *Giardia lamblia*) species. In co-infection research it has recently been recommended to consider a broad characterization of the pathogen community [25]. Thus, virus, bacteria and protozoa species richness was also included in the PLS’s X block. In addition, due to the previously mentioned seasonal variability in the incidence of acute diarrhea, the season (0 for dry and 1 for wet) was also included as a covariate in the explanatory block. Our response block (PLS’s Y component), however, described the severity of gastroenteritis including both the MNBS and MNLS per 24-h period.

The significance of PLS models was assessed using the Stone-Geisser’s Q2 test, a cross-validation redundancy measure created to evaluate the predictive significance of exogenous variables. Values greater than 0.0975 indicate that the exogenous variables are statistically significant, whereas values below this threshold reveal no significance. In a second step, MNBS per 24 h was finely excluded from our PLRS model because it low contribution to the PLS’s Y block (Stone-Geisser’s Q2 = -0.0014). Finally, the percentage of observed MNLS variability explained by the enteric pathogen block was also estimated.

For the PLS analysis, plspm library version 0.4.1 was used [56] and epiR library version 0.9–74 was used to calculate prevalences at 95 % CIs [57]. All analyses were performed using R version 3. 2.5 [51].

#### Probability of co-occurrence of enteric pathogens

We also explored whether co-infecting enteric pathogens detected in sick children (>4 liquid stools/24 h) were positive, negative, or randomly associated, using an individual pairwise co-occurrence approach [58]. In our case, data were organized as the presence-absence 8 × 715 (row × columns) matrix, in which each row represented a pathogen species and each column represented a child suffering from gastroenteritis. In this matrix, ‘1’ indicated that a species was present in a particular child and ‘0’ indicated that a species was absent. This method examines co-occurrence species by species to determine whether a particular pair of species is aggregated, segregated, or random in occurrence. The expected number of co-occurrences between pairs was obtained through randomization of enteric pathogens species in the studied children. For this analysis, only children with more than five liquid stools within 24 h were considered. Co-occurrence analysis was performed using “cooccur” package 1.3 version [59].

## Results

During the study period, a total of 1020 stool samples were collected from healthy (*n* = 170, normal stools) and sick (*n* = 850, liquid, mucousy, or bloody stools) children aged between one month and five years (mean age = 15 months). The sex ratio (male/female) in the group who had diarrhea was 1.002 (428/427), whereas it was 1.2 (90/75) in the asymptomatic group (χ^2^ = 0.94, *P* = 0.33).

In those who had diarrhea, vomiting (24.2 %, 206/850) was the most common clinical manifestation, followed by fever (13.5 %, 115/850) and dehydration (6.3 %, 54/850). In terms of stool types, 5.1 % (43/850) were bloody, 43 % were mucousy, and 52 % were liquid. In the group of sick children, the average number of liquid stools was 6.6 in a 24 h period (min = 4, max = 30).

### Single enteric pathogen infections

Prevalences at 95 % *CI*s and associated ORs (95 % *CI*s) for single infections and co-infections with enteric pathogens for both case and control children are shown in Table 2.

**Table 2:** Single infections and co-infections with enteric pathogens among asymptomatic and diarrheic children aged below five years in Kunming, southwest China

Single infections							
Infections	Dry season		Wet season		Total		OR
	Case	Control	Case	Control	Case	Control	
Viruses							
RVA	19.8	5.2	15.5	1.7	26.5	5.8	4.3 *
	(16.5 – 23.4)	(1.9 – 1.1)	(11.7 – 20.1)	(0.04 – 9.5)	(23.5 – 29.5)	(2.8 – 10.5)	(2.4 – 9.3)
NoV GII	3.5	2.6	6.1	5.3	4.4	3.5	1.2
	(2.1 – 5.4)	(0.5 – 7.4)	(3.6 – 9.2)	(1.1 – 14.83)	(3.1 – 6.1)	(1.3 – 7.5)	(0.5 – 3.3)
Protozoa							
*B. hominis*	1.3	0.8	6.3	--	3.1	0.5	4.7*
	(0.5 – 262)	(0.02 – 4.7)	(3.9 – 9.6)		(2.1 – 4.5)	(14e-05 – 3.2)	(1.01 – 112)
*Cryptosporidium* spp.	--	--	0.3	--	0.1	--	0.19
			(8.38e-05 – 1.7)		(2.9e-05 – 0.6)		(0.02 – 14.8)
Co-infections							
Virus-Virus							
RVA-NoV GII	5.2	--	2.8	3.5	4.4	1.1	3.2
	(3.5 – 7.4)		(1.3 – 5.3)	(0.4 – 12.3)	(3.1 – 6.1)	(0.1 – 4.1)	(0.9 – 21.2)
Bacteria-Virus							
DEC- RVA	2.8	--	1.5	--	2.3	--	3.9
	(1.5 – 4.5)		(0.5 – 3.6)		(1.4 – 3.6)		(0.4 – 136.5)
DEC-NoV GII	0.7	--	1.9	--	1.1	--	1.9
	(0.2 – 1.9)		(0.7 – 4.1)		(0.5 – 2.1)		(0.24 – 72.2)

Prevalences at 95 % CIs for single infections and co-infections with enteric pathogens among case (at least five stools/24 h) and control children aged below five years in Kunming, southwest China. Children were sampled during the wet (May to October, *n* = 371) and dry (November to April, *n* = 649) seasons. Odd ratios between case (*n* = 850) and control (*n* = 170) children were estimated at 95 % CIs. The asterisk (*) indicates statistical differences (calculated using the chi-square or Fisher’s exact tests) in pathogen prevalence between case and control children in both seasons. The “- -” symbol indicates that no cases were detected. Infections with various DEC subtypes (i.e., EAEC, EPEC, EAEC, EHEC, and ETEC) were combined into a single category (DEC). Only co-infections found in at least 10 individuals (1 % of children) have been shown

At least one enteric pathogen was found in 397 children (46.7 %) with acute gastroenteritis and in 23 healthy children (13.5 %). However, no enteric pathogen was detected in 453 children (53.3 %) who had diarrhea. Regarding single infections, the most common types in all children were caused by a virus species, followed by protozoan species. Single infections with bacterial species were not observed. *Shigella* spp., *Vibrio parahaemolyticus*, *Vibrio cholerae*, or *Plesiomonas* spp. were not detected in our study at all.

The most common single viral infection was due to RVA (26.5 % in case and 5.8 % in control children), followed by NoV GII (4.4 % in case and 3.5 % in control children). Though both viruses were more frequently found in sick children than in the asymptomatic controls (*OR*_RVA_ = 4.3, *OR*_NoV GII_ = 1.2), statistical differences in prevalences between cases and controls were only observed for RVA infections (χ^2^ = 32.7, *P* < 0.01). These case-control differences were detected in both seasons (χ^2^ = 47.3, *P* < 0.01 in wet season; χ^2^ = 95.7, *P* < 0.01 in dry season).

Along the same lines, NoV GII prevalence was 1.2 higher in case (4.4 %) children than in their asymptomatic counterparts (3.5 %). However, no statistical differences were observed between case and control children in any period of the year. Other viruses such as SaV, As, and Ad were only observed as co-infections with other enteric pathogens.

Regarding protozoan species, *B. hominis* was the most commonly observed enteric parasitic infection (3.1 % in cases and 0.5 % in controls, *OR* = 4.7, 95 % *CI*: 1.01–112.0). Although *B. hominis* was more frequently detected during the wet season, no statistical differences in the prevalence between cases and controls were observed between seasons. Single infection with *Cryptosporidium* spp. was uncommon and only detected during the wet season. No statistical differences were observed in *Cryptosporidium* spp. prevalences between sick and healthy children. Finally, *Giardia lamblia* infection was not observed in our study sample at all.

### Co-infections with enteric pathogens

Co-infection with two enteric pathogens was detected only in 5.3 % of asymptomatic children (9/170), but in 20.1 % (171/850) of children with acute gastroenteritis. Though 16 different types of co-infections involving virus-virus, virus-bacterial, virus-protozoan, and bacterial-virus species were observed in the children suffering from acute gastroenteritis, the first two types accounted for 71 % of all cases (see Table 4). The only bacteria-bacteria co-infections observed were DEC subtypes. RVA-NoV GII was the most common co-infection detected in 4.4 % of case and 1.1 % of control children. However, statistical differences in RVA-NoV GII co-infection prevalences between case and control children were not observed.

In decreasing order of importance, DEC-RVA (2.3 % of cases) and DEC-NoV GII (1.1 % of cases, see Table 2) co-infections were also observed in sick children. Contrary to what was observed in terms of single infections, co-infections in asymptomatic children were unusual; only RVA-NoV GII co-infections were observed (see Table 2). In line with the single infections, co-infections with enteric pathogens seemed to be more prevalent during the dry season (e.g., RVA-NoV GII, see Table 2). The seasonal differences, however, were not statistically significant.

### Impact of co-infection on the severity of diarrhea

**Fig. 1:**
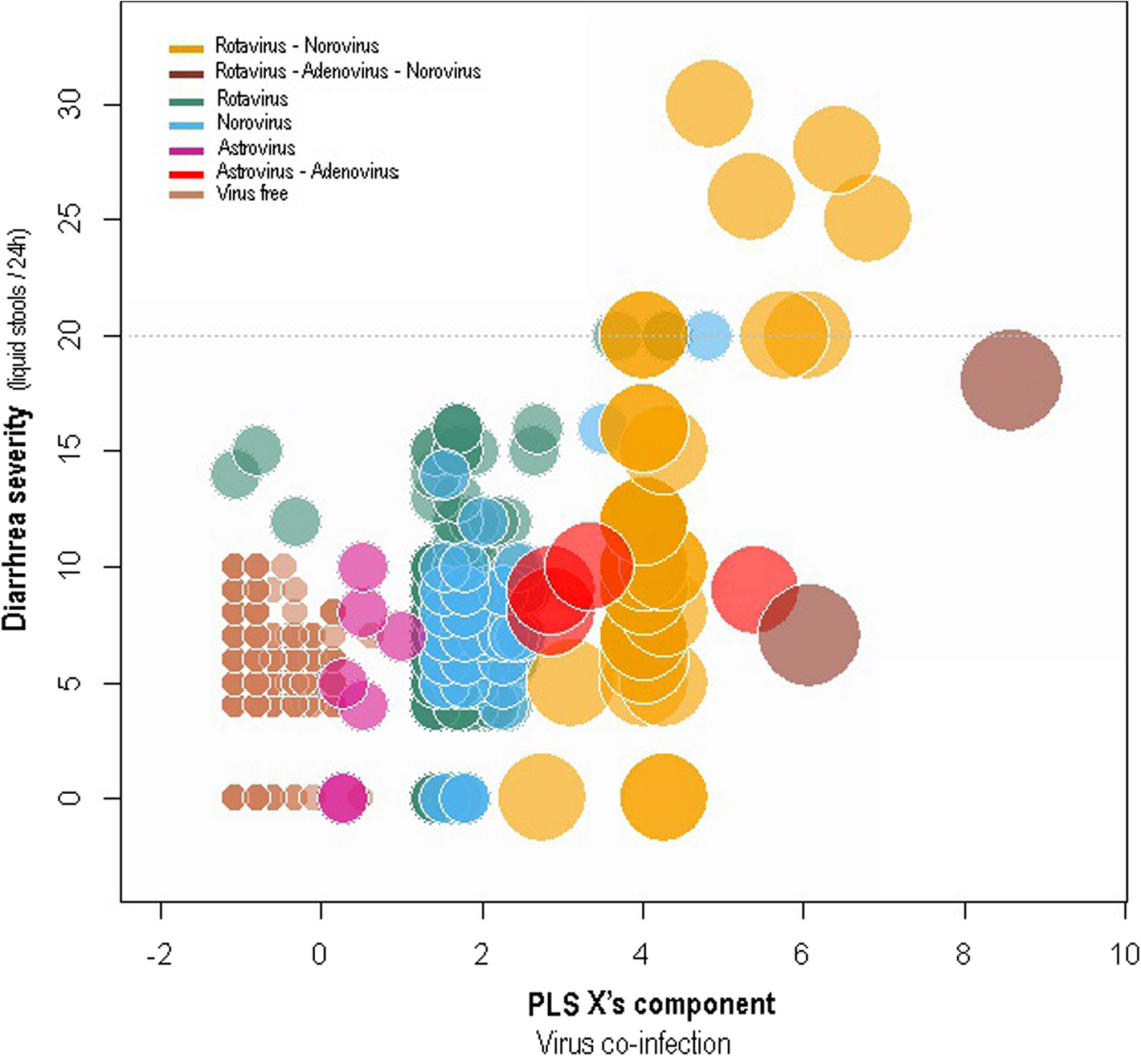
Bubble chart showing the relationship between PLS X’s component, representing the enteric pathogen community, and the severity of diarrhea (MNLS per 24 h) in 850 children aged below five years in Kunming, southwest China. In the PLS X’s component, virus richness, RVA (here shown as rotavirus), and NoV GII (shown as norovirus) were the most important variables of enteric pathogen PLS X’s component. On the other hand, diarrhea severity (response variable) was represented by the MNLS per 24 h. The initial set of variables representing the X’s component can be seen in Table 3. Bubble size indicates virus richness that ranged from 0 (virus free) to three virus types (children co-infected with RVA, Ad, and NoV). Bubble color, however, indicates a specific co-infection. The horizontal grey dotted line shows the 90th percentile

According to our PLS analyses, 23.8 % of observed variability of diarrhea severity in children was explained by co-infection with enteric pathogens. Most (93.4 %) of the X’s component variance was due to virus richness (43.2 %), and RVA (31.4 %) and NoV GII (18.8 %) infections (see Table 3). According to the cross-correlation values, however, virus richness was clearly the main factor linked to the severity of diarrhea. Interestingly, the three main virus infections in children with acute gastroenteritis (i.e., RVA, NoV GII, and Ad, see Table 3) covaried positively with diarrhea severity. In fact, as shown in Fig. 1, children with 20 or more liquid stools per 24 h were co-infected with combinations of the previously mentioned viruses. Other viral, bacterial, and protozoa infections had little effect on the severity of gastroenteritis. The PLS also revealed that the severity of gastroenteritis does not differ between seasons. In fact, the MNLS per day was six both in dry and wet season.

**Table 3:** Predictor weights of the PLS model explaining the severity of acute diarrhea in children aged below five years in Kunming, southwest China

Predictor variables	Loads	Weights	Percent	Cross-correlation
**Virus richness**	0.68	0.66	43.2	0.95
**RVA**	0.54	0.56	31.4	0.61
**NoV GII**	0.45	0.43	18.8	0.42
Ad	0.14	0.14	2.0	0.04
*B. hominis*	0.05	0.09	0.8	0.0
Protozoa richness	0.05	0.09	0.8	0.0
Bacteria richness	0.09	0.08	0.7	0.02
DEC	0.07	0.07	0.5	0.01
NTS	0.06	0.04	0.2	0.01
As	0.11	0.02	0.0	0.02
*Cryptosporidium* spp.	0.0	0.01	0.0	0.0
SaV	0.05	-0.01	0.0	0.0

Note: Predictor weights represent the contribution of pathogen to the PLS X’s component. Pathogens explaining more than 10 % of the observed PLS X’s block variability are shown in bold type. Cross-correlations represent the correlations between each pathogen and the MNLS per 24 h (diarrhea severity). Virus, protozoa, and bacteria richness signify the maximum number of species from each group detected in a child. Seasons have been defined as two periods: wet (May to October) and dry (November to April)

### Co-occurrence of enteric pathogens in sick children

**Fig. 2:**
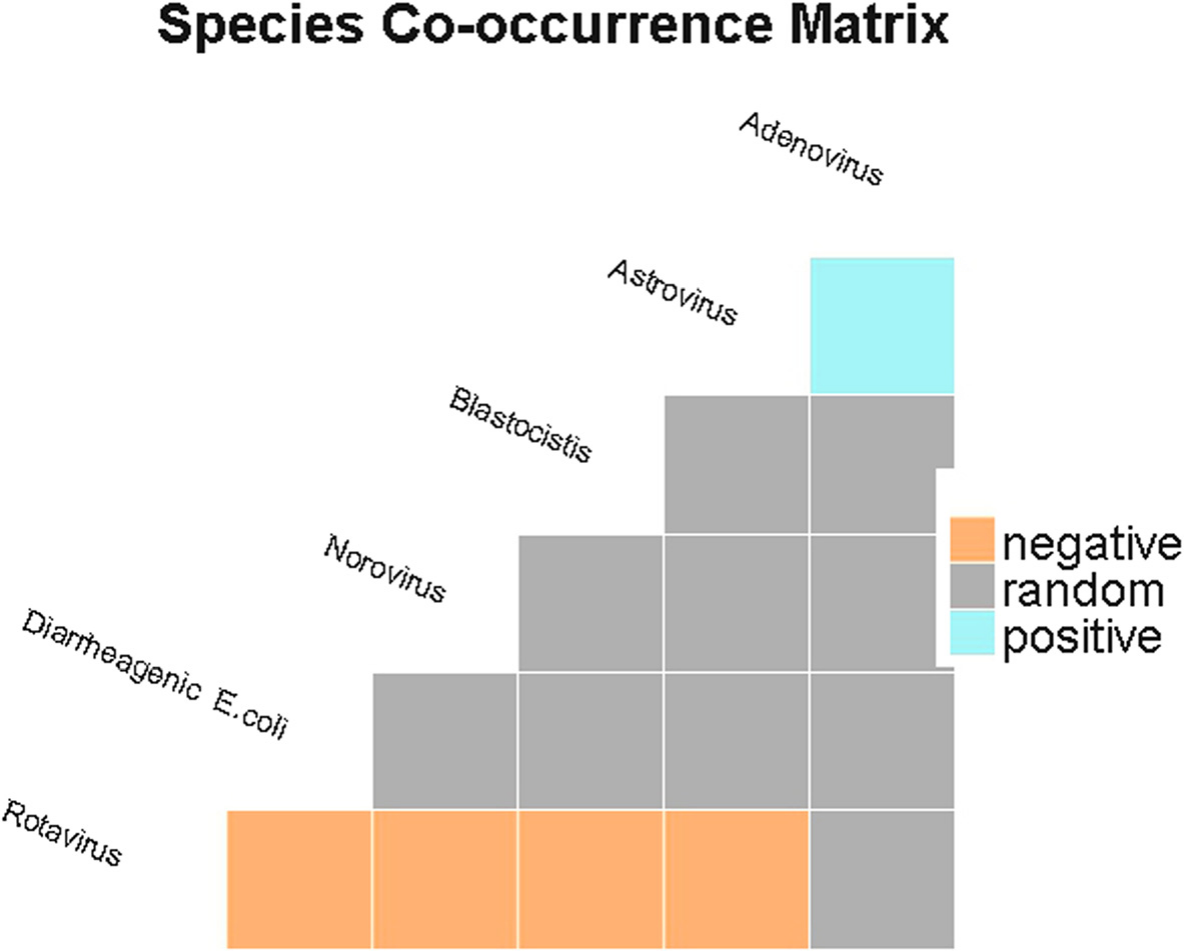
Heatmap showing associations between significant enteric pathogen species determined using the probabilistic co-occurrence model for enteric pathogens detected in 850 children (<5 years) suffering from acute diarrhea in Kunming, southwest China. The column and row represent pairwise relationship among two enteric pathogens. Boxes in grey color indicate random co-occurrences, orange boxes indicates associations were less common than expected by chance

According to our co-occurrence analysis, 82.1 % (23/28) of the classifiable species pairs showed random associations. Negative co-occurrences were found in 17.4 % (4/23) of children, whereas positive co-occurrences were found in only 4.3 % (1/23). Among the four significantly negative co-occurrences, only two (RVA-NoV GII and DEC-RVA) were found in at least 20 % of the children (see Table 4). Though, DEC-NoV GII co-infection was detected in at least 4 % of the sick children, this species association was truly random. Interestingly, all associations involving RVA (i.e., RVA-NoV GII, RVA-DEC, RVA-Bh, and RVA-As) occurred less frequently than expected by chance (see Table 4 and Fig. 2). Finally, though the As-Ad co-infection occurred more frequently than expected by chance, it was uncommon (1 % of children).

**Table 4:**
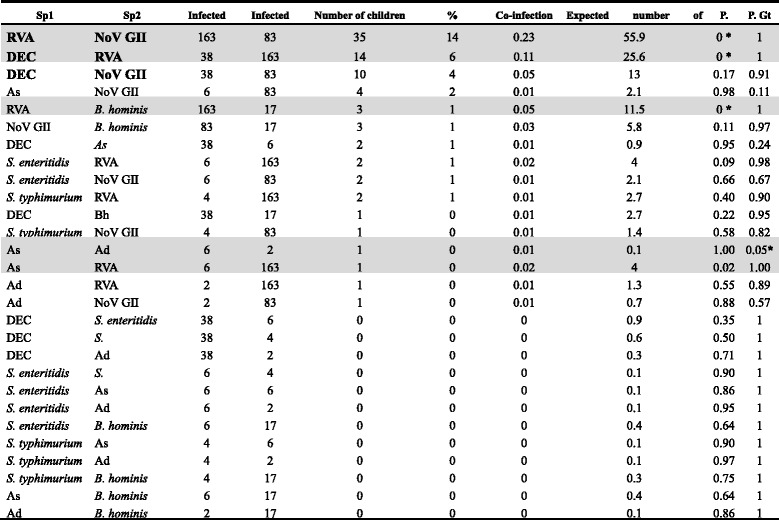
Pairwise probability table showing single infections and co-infections with enteric pathogens in diarrheic children aged below five years in Kunming, southwest China

Note: For this analysis, only children with more than five liquid stools within 24 h were considered. “Infected with sp1” indicates the number of children infected with one of the species shown in the first column. The same rationale is applied for “Infected with sp2”. “%” indicates the percentage of children co-infected by a particular pair of pathogens. Co-infection probability is the probability of a child suffering from a particular co-infection. The expected number of co-infected children is the theoretical number of children with co-infections. P. Lt and P. Gt represent the probabilities that those species could co-occur less (P. Lt) than or greater (P. Gt) than what is observed in our data, respectively. They can be interpreted as *p*-values, thus indicating significance levels for negative and positive co-occurrence patterns. Significant *p*-values have been indicated by an asterisk “*”. Co-infection types observed in at least 10 children have been bolded. Shaded rows indicate co-infections that do not co-occur randomly

## Discussion

Most research on acute diarrhea in children has mainly focused on the effects of single pathogen infections (e.g., RVA [60], NoV [7]), or infections with pathogen groups (e.g., enterobacteria [35]). Limited research that considers the broad representation of the enteric pathogen community in children has been conducted [61]. This study is one of the few laboratory-based investigations on diarrheal illness involving co-infections with viral, bacteria, and parasitic agents in children.

As we have seen and in line with previous research [62], the observed overall prevalence of enteric causative agents in diarrhea cases was as high as it was in the healthy controls making difficult to determine the true etiological role of enteric pathogens in children suffering from acute gastroenteritis. Diarrhea severity in sick children, however, appears to be linked to specific co-infections as we will see below.

### Single- and co-infections with enteric pathogens in case and control children

In any case, it appears clear that viral and bacterial pathogens are the most common causative agents in diarrhea cases in Yunnan. Overwhelmingly RVA causes severe diarrhea in children, and high numbers of diarrhea deaths worldwide are contributed to this virus [2, 6, 60]. Along the same lines, RVA was the most common viral pathogen detected in children suffering from acute gastroenteritis in Yunnan. Though results of other studies concur with this finding [63, 64]. Detection rates of RVA here were slightly lower than that previously reported in other regions of China [12]. The prevalence of RVA in diarrhea cases was different, which might be attributed to the different source of diarrhea cases, the cases of present were all being outpatients, but cases of nationwide surveillance of China were all inpatient children. Another study also showed that the prevalence of RVA in outpatient cases was lower than that in inpatient cases [63]. In comparison with other countries, RVA prevalence in Yunnan is close to the detection rate of RVA in children with diarrhea was as high as that in some developing Asian and Sub-Saharan African countries [63, 65], and was also similar to the prevalence of RVA in countries in the pre-vaccine period [66]. However, if a RVA vaccine is part of a country’s immunization program, the prevalence of RVA in that country/region is low, regardless of what the social and economic development level is [66, 67]. Hence, a safe, effective, and affordable RVA vaccine is an efficacy measure for reducing infection with RVA and associated death, especially in low-income countries.

Single NoV GII infections were the second most common pathogen detected in the present study. In recent years, use of PCR [8] has shown how NoV GII play a major role in sporadic diarrheal illnesses [7]. Fish and other meat products can become easily contaminated with NoV, being probably the main source of NoV contamination in Yunnan. Single infections with SaV, AS and Ad were not observed in sick children but in co-infection with other enteric pathogens. As these virus are commonly considered as common causes of mild diarrhea in children as confirmed in our survey [68, 69].

Prevalence of enteric bacterial pathogens in sick children was very low in Yunnan province. In fact, single infections with bacterial species were not observed, and DEC or NTS occurred in co-infection with other enteric pathogens only. In comparison with enteric viral pathogens DEC or NTS appeared to play a secondary role in children with acute gastroenteritis. Though these results are inconsistent with previous research made in China [11, 12], as well as in other developing countries [14]. Accordingly, it appears that enteric bacterial pathogens widely exist in developing regions and that children are tolerable to being exposed to enteric pathogens for long periods. Hence, it would be suggested that these enteric bacteria work in co-infection with other enteric pathogens to cause and aggravate diarrhea disease.

In other order of things, diagnostic method used to detect DEC in many studies has been the serum agglutination method which is characterized by a low sensitivity and specificity [11]. In consequence, DEC prevalence would have been seriously underestimated. Diagnostic methods such as PCR can shorten the diagnosis time and more precisely distinguish the subtype of DEC, which can provide the reference to treat diarrhea with DEC infection. However, due to issues such as restrictions on funding and the capacity of laboratory staff [11, 12], the PCR method has not become the conventional detection method worldwide, including in China. Hence, it is imperative to develop a fast and cheap diagnostic kit with a high sensitivity.

In line with previous reports [70], *B. hominis* was the most common protozoan enteric pathogen in diarrhea cases in Yunnan. Though *B. hominis* is a ubiquitous parasite with worldwide distribution [71, 72], there is still great dispute on its pathogenicity [71]. The same occurred with *Cryptosporidium* spp. and *Giardia lamblia*, two other well know pathogenic diarrhea agents worldwide [73, 74], but with low relevance in our study. As observed for enteric bacteria, reports linking protozoa to gastroenteritis have not been performed under a co-infection perspective, being difficult to clarify whether or not other enteric pathogens were also involved in the clinical outcome.

As found for single infections, prevalence of co-infection with two enteric pathogens was slightly higher in diarrhea cases (*OR* = 1.9 for DEC-NoV GII, whereas 3.9 for DEC- RVA co-infections) than in asymptomatic controls. Actually, these co-infections can be also observed in asymptomatic patients [13, 75], probably indicating that when one intestinal pathogen infected the body the infection rate of another pathogen also rises [76]. On the other hand, these foodborne pathogens can be found in the same contaminated foods or beverages increasing the likelihood of mixed infections in risk areas. In fact, co-infections with multiple enteric pathogens occur mainly in regions with poor sanitary conditions in the environment, and poor quality of food and drinking water contributing to the widespread of microorganisms [77].

### Co-infections and diarrhea severity

Though co-infections are also detected in control children, specific virus-virus co-infections such as RVA-NoV GII, or RVA-NoV GII-Ad clearly aggravated diarrhea symptoms. There are two reasons that could possibly explain this. On the one hand, the likelihood of infection with both pathogens can actually increase by the fact that both viruses are among the most prevalent enteric pathogens in Yunnan. On the other hand, to a certain extent, the comprised means of co-infections also depend on the interaction between the intestinal pathogens as previously observed [61, 78].

In any case, there is no doubt that co-infections have a deleterious effect on health exacerbating infection outcome in humans [26]. The interpretation for this is similar to that given for the course of diarrhea. The two pathogens have synergism and the pathogenic potential of each organism appears to be enhanced during co-infection [61], Hence, co-infection will aggravate the diarrhea as previously suggested [79].

### Co-occurrence with enteric pathogens in sick children

Curiously, the key characters driving acute diarrhea in Yunnan, e.g., RVA-NoV GII, DEC-RVA or RVA-*B. hominis* co-occurred at a frequency lower than expected by chance. Only one co-infection pair As-Ad followed the opposite pattern, but this pathogen combination was extremely rare. There are not evidences for cross-protective immunity for any of the previously mentioned co-infection pairs (e.g., see [80], so the most plausible explanation to explain this pattern is that RVA or NoV GII alone are causes of hospitalization diminishing the risk of co-infection with a second pathogen.

### Limitations of study

This study had several limitations [81, 82]. Firstly, it was conducted only in an urban area, and the pathogenic spectrum did not represent the overall and exactly all local cases. Secondly, the diarrhea cases were recruited from hospital outpatient departments. Hospitalized patients and diarrhea cases who did not attend a medical institution were not recruited, which might have caused bias. Thirdly, DAEC and helminthes were not detected in this study. Fourthly, enteric protozoa were not detected with microscopy. Therefore, further research on the severity of diarrhea, which considers a broader enteric pathogen community, in urban, rural, outpatient, and inpatient children will need to be conducted.

## Conclusions

Although it is clear that RVA has an overwhelming impact on diarrhea in children, the potential for pathogenesis to be strengthened in the presence of a rotavirus co-infection, which amplifies the need for RVA vaccination. In addition, further studies are needed to reveal the comprehensive and molecular biological mechanisms of enteric pathogens resulting in their interactions.

## Abbreviations

Ad, adenovirus; As, astrovirus; *B. hominis*, *blastocystis hominis*; CI, confidence interval; DAEC, diffusely adherent *E. coli*; DEC, diarrheagenic *E. coli*; DNA, deoxyribonucleic acid; EAEC, enteroaggregative *E. coli*; EHEC, enterohemorrhagic *E. coli*; EIEC, enteroinvasive *E. coli*; EPEC, enteropathogenic *E. coli*; ETEC, enterotoxigenic *E. coli*; MAC, MacConkey agar; MNBS, maximum number of bloody stools; MNLS, maximum number of liquid stools; NoV, norovirus; NTS, non-typhoidal *Salmonella*; OR, odds ratio; PCR, polymerase chain reaction; PLS, partial least square; qPCR, real-time quantitative polymerase chain reaction; RNA, ribonucleic acid; RT-PCR, reverse transcriptase polymerase chain reaction; RVA, rotavirus group A; SaV, *Sapovirus*; WHO, World Health Organization

## Additional file

Additional file 1: Multilingual abstracts in the six official working languages of the United Nations. (PDF 904 kb)
