# Supplementary material for: Impact of co-infections with enteric pathogens on children suffering from acute diarrhea in southwest China
Source: Infect Dis Poverty. 2016 Jun 27;5:64. doi: 10.1186/s40249-016-0157-2 (PMC4922062; doi:10.1186/s40249-016-0157-2)
Supplement: Additional file 1: — Multilingual abstracts in the six official working languages of the United Nations. (PDF 904 kb) [file 40249_2016_157_MOESM1_ESM.pdf]

Translation of the abstract into the six official working languages of the United Nations

تأثير العدوى المصاحبة مع الاسباب المرضية المعوية على الاطفال الذين يعانو من اسهال حادّ في الجنوب الغربي للصين

Shun-Xian Zhang, Yong-Ming Zhou, Wen Xu, Li-Guang Tian, Jia-Xu Chen, Shao-Hong Chen, Zhi-Sheng Dang, Wen-Peng Gu, Jian-Wen Yin, Emmanuel Serrano, Xiao-Nong Zhou

شون شين تشانغ، يونغ مينغ تشو، ون شو، لي قوانغ تيان، جيا شو تشن، شاو هونغ تشن، تشي شنغ دانغ، ون بينغ قو، جيان ون يين، ايمانويل سيرانو، شياو نونغ تشو

ملخص

لمحة: الاسهال الحاد هو مشكل صحي عالمي ناتج عن المَراضَة العالية ومعدل الوفيات عند الاطفال. لقد اقترح بأن عدوى المرافقة للاسباب المرضية المعوية تلعب دورًا هام في التهاب المعدة والامعاء لكن معظم مجهودات البحث ركزت فقط على فئة صغيرة من العيّنات رادعة لجموعات قليلة من السبب المرضي. تهدف هذه الدراسة إلى تقيّم تاثير العدوى المرافقة مع الأسباب المرضية عند الاطفال تحث خمس سنوات الذين يعانو من اسهال حاد في الجنوب الغربي للصين.

الاسلوب:إخترنا مجموعة مواضيع 1020 (850 حالات اسهال و 170مرقبات صحّية) من أربع رصد أو الأمراض المعدية مستشفيات في كيومينق ومقاطعة يانن في جنوب غرب الصين ما بين حزيران 2014 إلى يوليو 2015. جُمعت عينات البراز لكشف الفيروسات (مجموعة الفَيروسَةُ العَجَلِيَّة (A) RVA وفيروس القيء NoV و فيروس صابونيSaV وفيروس أسترو النجمي وفيروس الغُدَّانِيَّة: Ad. وسبع بكتيرات (بكتيريا الاسهال المُورثية الإشريكية القلونيةDEC وبكتيريا السَّلْمونيلَة الغير تيفيّة NTS وبكتيريا الشِّيغِيلَّة سبا وبكتيريا ضَمَّةُ الكُوليرا وبكتيريا الضَّمَّةُ نظيرةُ الحالَّةِ للدَّم وبكتيريا سبا الغازية وبكتيريا سبا القَوْرَبِيَّة) وثلاث عينات متعلق بالأَوَالِي (خَفِيَّةُ الأَبْواغSPP، و الجِيارْدِيَّةُ اللَّمْبِلِيَّة و المُتَبَرْعِمَةُ الكيسِيَّةُ البَشَرِيَّة) باستخدام معيار دقيق بيولوجي وطرق جزئية. حللت المعطيات باستخدام تقنية الانحدار بطريقة المربّعات الصغيرة جُزئيًا واِختبا خِي مُرَبَّع.

النتائج: على الاقل واحد من الاسباب المرضية المعوية إكْتُشِفَ في (n=397) 46.7% لحالات التهاب المعدة الحاد و (n=23) للمراقبات الصحية. عدوى واحدة بفيروسRVA كانت مرتبطة مع الاسهال الحاد(26.5% *vs.* 5.8%, $P<0.05$). التحكم في عدوى واحدة بالمُتَبَرْعِمَةُ الكيسِيَّةُ البَشَرِيَّة في حالات الاسهال كانت مرتفعة في المراقبات الصحية (3.1% *vs.* 0.5%, OR=4.7, 95%CI: 1.01–112.0). وعدوى واحد ب وفيروس القيء NoV GII لم تكن مرتبطة مع الاسهال (4.4% *vs.* 3.5%, OR=1.2, 95%CI: 0.5–3.3). عدوى واحدة بعينات البكتيرية لم ترصد. التحكم في العدوى المصاحبة مع سببين مرضية معوية في حالات الاسهال كانت عالية من الاطفال غير معديين(20.1% *vs.* 5.3%, $P<0.05$). كان فيروس RVA-NoV GII الاكثر شيوعًا للعدوى المصاحبة في الاطفال غير معديين (4.4%) مع تشديد تَعَصُّب الاسهال.

الاستنتاجات: بالرغم انه من الواضح أن فيروس RVA لديه تأثير كبير في امراض الاسهمال عند الاطفال. تظهر العدوى المصاحبة مع الاسباب المرضية المعوية إيضًا زيادة تهجم الاسهال. هذه النتائج يجب أن تتخذ كدليل من أجل خدمات الصحة العمومية عند تخطيط وتطوير برامج المداخلات.

Translated from English version into Arabic by Ghania Khalifa, through

TRANSLATORS
WITHOUT BORDERS

中国西南地区 5 岁以下急性腹泻患儿中肠道病原体混合感染的影响

张顺先，周永明，徐闻，田利光，陈家旭，陈韶红，党志胜，古文鹏，尹建雯, Emmanuel Serrano，周晓农

摘要：

引言：急性腹泻是全球重要的公共卫生问题，每年有很多儿童发生腹泻，因腹泻死亡的儿童也有很多。肠道病原混合感染在腹泻发生过程中起重要作用，但很多腹泻研究涉及的病原体范围较小。本研究的目的是

探索我国西南地区 5 岁以下急性腹泻儿童中肠道病原体混合感染的流行特征。

方法：从 2014 年 7 月 1 号到 2015 年 6 月 30 号，在云南省昆明市选择 4 家哨点医院作为研究现场，共纳入 1 020 名研究对象（850 名腹泻病例和 170 名非腹泻病例），收集粪便样本，使用标准的微生物检测方法检测粪便样本中多种肠道病原体：5 种病毒（轮状病毒、诺如病毒、扎如病毒、星病毒和腺病毒）、7 种细菌（致腹泻性大肠杆菌、非典型性沙门菌、志贺杆菌、霍乱弧菌、副溶血性弧菌、邻单胞菌和气单胞菌）和 3 种原虫（隐孢子虫、蓝氏贾第鞭毛虫和人芽囊原虫）。数据分析采用偏最小二乘回归法和卡方检验。

结果：肠道病原体总检出率在腹泻病例中高于非腹泻病例（46.7%，n=397；13.5%，n=23；χ^2=64.4, P<0.05），轮状病毒是腹泻相关的病原体（26.5% vs. 5.8%, P<0.05），人芽囊原虫也是腹泻相关的病原体（3.1% vs. 0.5%, OR=4.7, 95%CI: 1.01–112.0），而诺如病毒 GII 不是腹泻相关的病原体（(4.4% vs. 3.5%, OR=1.2, 95%CI: 0.5–3.3）。腹泻病例中细菌性病原体都和其他病原体以混合感染的形式存在。混合感染的总检出率在腹泻病例中高于非腹泻病例（20.1%，n=171；5.3%，n=9；χ^2 = 26.5, P<0.05）。腹泻病例中轮状病毒和诺如病毒 GII 的混合感染率为 4.4%，且混合感染加重腹泻。

结论：尽管轮状病毒是 5 岁以下儿童腹泻最重要的病原体，但它和其他病原体的混合感染加重腹泻。本研究的发现可为公共卫生部门制定腹泻防控措施提供科学参考。

Translated from English version into Chinese by Zhang Shun-Xian

Impact des co-infections par des agents pathogènes entériques chez les enfants souffrant de diarrhée aiguë dans le Sud-ouest de la Chine

Shun-Xian Zhang, Yong-Ming Zhou, Wen Xu, Li-Guang Tian, Jia-Xu Chen, Shao-Hong Chen, Zhi-Sheng Dang, Wen-Peng Gu, Jian-Wen Yin, Emmanuel Serrano, Xiao-Nong Zhou

Résumé

Contexte : Les diarrhées aiguës de l'enfant constituent un problème mondial, cause d'une morbidité et d'une mortalité infantiles importantes. Certains auteurs ont suggéré que des co-infections par des pathogènes entériques jouaient un rôle important dans ces gastroentérites, mais les travaux de recherche se sont souvent limités à un petit nombre d'espèces appartenant à quelques groupes. La présente étude visait à évaluer l'impact des co-infections par des pathogènes entériques très divers chez des enfants âgés de moins de cinq ans souffrant de gastroentérite dans le Sud-ouest de la Chine.

Méthode : Au total, 1020 sujets (850 cas de diarrhée et 170 témoins en bonne santé) ont été sélectionnés dans quatre hôpitaux sentinelles de Kunming, dans la province du Yunnan, entre juin 2014 et juillet 2015. Des échantillons de selles ont été recueillis afin de rechercher cinq virus (rotavirus du groupe A [RVA], norovirus [NoV], sapovirus [SaV], astrovirus [As] et adénovirus [Ad]), sept bactéries (*Escherichia coli* diarrhéigènes [DEC] *Salmonella* non typhique [NTS], *Shigella* spp., *Vibrio cholerae*, *Vibrio parahaemolyticus*, *Aeromonas* spp. et *Plesiomonas* spp.) et trois protozoaires (*Cryptosporidium* spp., *Giardia lamblia* et *Blastocystis hominis*) par les méthodes microbiologiques et moléculaires standard. Les données ont été analysées par la méthode des moindres carrés partielle et le test de chi-deux.

Résultats : Au moins un pathogène entérique a été décelé dans 46,7 % (n=397) des cas de gastroentérite aiguë et 13,5 % (n=23) des témoins en bonne santé (χ^2=64,4, P<0,05). L'infection par le rotavirus A seul était associée à des diarrhées aiguës (26,5 % contre 5,8 %, P<0,05). La prévalence de l'infection par *B. hominis* seul était plus élevée parmi les cas de diarrhée que chez les témoins en bonne santé (3,1 % contre 0,5 %, OR=4,7, IC à 95 % : 1,01–112,0). L'infection isolée par NoV GII n'était pas associée à des diarrhées (4,4 % contre 3,5 %, OR=1,2, IC à 95 % : 0,5-3,3). Nous n'avons pas observé d'infections par une seule espèce bactérienne. La prévalence des co-

infections par deux pathogènes entériques était plus élevée dans les cas de diarrhée que parmi les enfants asymptomatiques (20,1 % contre 5,3 %, *P*<0,05). L'association du RVA et du NoV GII était la co-infection la plus fréquente chez les enfants symptomatiques (4,4 %) et elle était la cause de diarrhées aggravées.

Conclusions : Bien que le rôle prépondérant du RVA dans les maladies diarrhéiques infantiles soit évident, il s'avère que la sévérité des diarrhées est aggravée par les co-infections avec d'autres pathogènes entériques. Il serait utile que les services de santé publique tiennent compte de ces données dans la planification et l'élaboration des programmes d'intervention.

Translated from English version into French by Suzanne Assenat, through

Воздействие коинфицирования возбудителями кишечных заболеваний на детей, страдающих острой диареей в юго-западном Китае

Шунь-Сянь Чжан, Юн-Мин Чжоу, Вэнь Сюй, Ли-Гуан Тянь, Цзя-Сюй Чэнь, Шао-Хун Чэнь, Чжи-Шэн Дан, Вэнь-Пэн Гу, Цзянь-Вэнь Инь, Эммануэль Серрано, Сяо-Нун Чжоу

Реферат

История вопроса: Острая диарея представляет собой всемирную медицинскую проблему, характеризующуюся высоким уровнем заболеваемости и смертности у детей. Высказывались предположения, что немаловажное воздействие на течение гастроэнтерита оказывает коинфицирование возбудителями кишечных заболеваний, но большая часть исследований проводилась на небольшом количестве видов, принадлежащих к нескольким группам возбудителей. Данное исследование ставило перед собой цель определить воздействие коинфицирования широким спектром возбудителей кишечных заболеваний на детей в возрасте до пяти лет, страдающих острой диареей в юго-западном Китае.

Метод: Для исследования с июня 2014 г. по июль 2015 г. было отобрано 1020 участников (850 больных диареей и 170 здоровых контрольных участников) из четырех контрольных больниц в г. Куньмин провинции Юньнань в юго-западном Китае. Были собраны анализы кала для обнаружения с помощью стандартных микробиологических или молекулярных методов пяти вирусов (ротавирусы группы A (RVA); норовирус (NoV); *саповирус (*SaV); астровирус (As) и аденовирус (Ad)), семи бактерий (диарейная кишечная палочка *Escherichia coli*, DEC; не брюшнотифозная *сальмонелла* (NTS); *шигелла*; *холерный вибрион; парагемолитический вибрион; аэромонас* и *плезиомонас*) и трех видов простейших (*криптоспоридия*, *лямблия кишечная* и *бластоцистис хоминис* (*B. Hominis)*). Анализ данных производился с помощью регрессии методом дробных наименьших квадратов и теста хи-квадрат.

Результаты: В 46,7% (*n*=397) случаях острого гастроэнтерита и у 13,5% (*n*=23) здоровых контрольных участников (χ^2=64.4, *P*<0.05) был обнаружен хотя бы один возбудитель кишечных заболеваний. Была установлена связь одиночной инфекции ротавирусами группы A (RVA) с острой диареей (26.5% *vs.* 5.8%, *P*<0.05). Распространённость одиночного инфицирования *B. hominis* в случаях диареи была выше, чем у здоровых участников контрольной группы (3.1% *vs.* 0.5%, *OR*=4.7, 95% *CI* (*доверительный интервал при доверительной вероятности)*: 1,01–112,0). Одиночное инфицирование NoV GII не связывалось с диареей (4.4% *vs.* 3.5%, *OR*=1.2, 95% *CI*: 0,5–3,3). Одиночное бактериальное инфицирование не было отмечено.

Распространённость коинфицирования двумя возбудителями кишечных заболеваний в случаях диареи было выше, чем у пациентов, не обнаруживающих симптомов заболевания (20.1% *vs.* 5.3%, P<0.05). RVA-NoV GII являлся наиболее распространенным видом коинфицирования у детей с клиническими проявлениями (4.4%), что осложняло ход диареи.
Выводы: Установлено, что инфицирование ротавирусами группы A (RVA) оказывает сильнейшее воздействие на ход диареи у детей, кроме того коинфицирование другими возбудителями кишечных заболеваний также повышает серьезность этого заболевания. Результаты данного исследования должны использоваться общественными системами здравоохранения при разработке и планировании программ по борьбе с этими заболеваниями.

Translated from English version into Russian by Alena Hrybouskaya, through

Impacto de las coinfecciones con patógenos entéricos en los niños que sufren de diarrea aguda en el suroeste de China

Shun-Xian Zhang, Yong-Ming Zhou, Wen Xu, Li-Guang Tian, Jia-Xu Chen, Shao-Hong Chen, Zhi-Sheng Dang, Wen-Peng Gu, Jian-Wen Yin, Emmanuel Serrano, Xiao-Nong Zhou

Resumen
Antecedentes: La diarrea aguda es un problema de salud global, que resulta en una alta morbilidad y mortalidad en los niños. Se ha sugerido que coinfecciones entéricas patógenas desempeñan un papel importante en la gastroenteritis, pero la mayoría de los esfuerzos de investigación se han centrado únicamente en una pequeña gama de especies pertenecientes a algunos grupos de patógenos. Este estudio tuvo como objetivo evaluar el impacto de las coinfecciones con una amplia gama de patógenos entéricos en niños menores de cinco años que sufren de diarrea aguda en el sudoeste de China.
Método: Un total de 1 020 sujetos (850 casos de diarrea y 170 controles sanos) fueron seleccionados de cuatro hospitales centinela en Kunming, provincia de Yunnan, suroeste de China, de junio de 2014 a julio de 2015. Se recogieron muestras de heces para detectar cinco virus (rotavirus del grupo A, RVA; norovirus, NoV; *Sapovirus,* SaV; astrovirus, As; y adenovirus, Ad), siete spp. bacterianas (diarreogénica *Escherichia coli*, DEC; no tifoidea *Salmonela*, NTS; *Shigella*; *Vibrio cholera*; *Vibrio parahemolitico*; *Aeromonas* spp.; y *Plesiomonas* spp.), y tres especies de protozoos (*Cryptosporidium* spp., *Giardia lamblia*, and *Blastocystis hominis*, *B. hominis*) utilizando métodos microbiológicos y moleculares estándar. Los datos fueron analizados mediante la técnica de regresión parcial al cuadrado menor y la prueba de chi-cuadrado.
Resultados: Por lo menos un patógeno entérico se detectó en el 46,7% (*n*= 397) de los casos de gastroenteritis aguda y el 13,5% (*n*= 23) de los controles sanos (χ^2= 64,4, *P*<0,05). La infección simple con RVA se asoció con diarrea aguda (26,5% *vs.* 5,8%, *P*<0,05). La prevalencia de la infección simple con *B. hominis* en los casos de diarrea fue mayor que en los controles sanos (3,1% *vs.* 0,5%, *OR*= 4,7, 95%*CI*: 1,01 a 112,0). La infección simple con NoV GII no se asoció con diarrea (4,4% *vs.* 3,5%, *OR*= 1,2, 95%*CI*: 0,5-3,3). No se observaron infecciones simples con especies bacterianas. La prevalencia de coinfecciones con dos patógenos entéricos en los casos de diarrea fue mayor que en niños asintomáticos (20,1% *vs.* 5,3%, *PAG*<0,05). RVA-GII el NoV fue la coinfección m

ás común en los niños sintomáticos (4,4%), y esto agravaba la gravedad de la diarrea.

Conclusiones: Aunque está claro que RVA tiene un gran impacto en las enfermedades diarreicas en los niños, la coinfección con otros patógenos entéricos parece agravar también la severidad de la diarrea. Estos resultados deben servir como prueba para los servicios de salud pública en la planificación y desarrollo de programas de intervención.

Translated from English version into Spanish by María Diehn, through

TRANSLATORS
WITHOUT BORDERS
